# Inkjet-Printed Graphene Electrodes on a Plastic Armband for Mobile Electrocardiography

**DOI:** 10.1007/s10916-026-02357-6

**Published:** 2026-03-05

**Authors:** Saygun Guler, Seyed Sajjad Mirbakht, Melih Can Tasdelen, Burcu Arman Kuzubasoglu, Faruk Ballipinar, Murat Kaya Yapici

**Affiliations:** 1 Faculty of Engineering and Natural Sciences, Sabanci University, Orta Mah., Universite Cad., Istanbul, 34956 Türkiye; 2 Sabanci University Nanotechnology Research and Application Center (SUNUM), Sabanci University, Orta Mah., Universite Cad., Istanbul, 34480 Türkiye; 3 Department of Electrical and Computer Engineering, University of Washington, 185 Stevens Way, Seattle, WA 98195-2500 USA

**Keywords:** Inkjet printing, Graphene, Mobile health, ECG electrode, Flexible, Wearable, Plastic, Armband, Internet of things (IoT), Medical garment, Nanomaterials, Personalized medicine

## Abstract

Drop-on-demand inkjet printing has shown great potential for wearable health monitoring applications because of its ability to directly pattern on flexible substrates that can conform to curved surfaces such as the skin. Surface biopotential measurements such as electrocardiography is one such example requiring conductive electrodes that can be attached to skin to record the electrical activity of the heart, otherwise known as an electrocardiogram (ECG). Typical pre-gelled, silver/silver chloride (Ag/AgCl) electrodes; also known as ”wet electrodes”, are known to cause skin irritations with performance degradation over time, and therefore remain largely non-ideal especially in long-term, mobile heath monitoring scenarios. This paper reports, for the first time, the development of a single, fully inkjet-printed graphene-on-plastic monolithic wearable armband, whose performance was benchmarked against commercial Ag/AgCl electrodes during a 1-hour-long ECG recording with five participants. The inkjet-printed graphene-on-plastic armband displayed excellent ECG reception with a signal-to-noise ratio (SNR) of up to 4.2 dB higher than that of commercial electrodes.

**Supplementary Information:**

The online version contains supplementary material available at 10.1007/s10916-026-02357-6.

## Introduction

Patient monitoring research has drawn significant attention as the growing rise in diabetes and obesity incites the risk factors of cardiovascular diseases (CVD) around the world [1]. Electrocardiogram (ECG) is a ubiquitous test that can reveal vital information regarding the cardiovascular dynamics of a subject/patient [2]. Conventional sensors used in a typical ECG setting are usually Ag/AgCl based sticky disposable electrodes that are carefully placed on the skin to measure biopotential difference [3]. Although these wet electrodes have proved highly accurate and suitable for medical purposes, numerous complications have been reported in the literature, especially with oversensitive skin types [4]. Such limitations have led to a rapid growth in dry electrode research [5]. These types of electrodes do not require laborious skin preparation or viscous gels, and their conformity to uneven skin surfaces makes them feasible for continuous (long-term) monitoring. Utilizing dry electrodes, researchers have focused on developing wearable alternatives to medical-grade measurement tools with the help of flexible electronics, along with various fabrication techniques aiming to minimize the electrode-skin impedance and motion artifacts while maximizing signal quality and comfort [6]. These bio-potential sensor studies include but are not limited to those focusing on micro-structures built on flexible substrates [7], conductive textiles [8], and printable materials [9].

Depending on their placement, wearable electrodes can be taxonomically surveyed as invasive, capacitive, or surface types. Invasive types of electrodes penetrate through the stratum corneum of the epidermis via many tiny stingers. Advancements in semiconductor technology have made it possible to process layers on a micro-scale level, thus allowing for the formation of needle-like arrays on flexible substrates [10]. Though working on a micro-scale requires expert-level demanding effort in a clean-room environment, these structures have been proven to adapt better to mobility than commercial electrodes [11]. Capacitive electrodes, on the other hand, are noninvasive and can sense bio-potential differences in the presence of a dielectric gap (i.e., clothes) between the conductive surface and the skin [12]. The severe negative influence of motion artifacts in capacitive electrodes has been targeted previously by several innovative studies [13, 14]. Unlike the invasive types, surface electrodes have less risk in terms of skin irritation, and they are less susceptible to motion than capacitive ones. Numerous material combinations have been proposed as insulator substrates (e.g., polydimethylsiloxane, PDMS; thermoplastic polyurethane, TPU; polyvinyl butyral, PVB) [15], and conductive sensing bits (e.g., silver, copper, graphene) [16] for comfortable and long-term wearable ECG applications.

Because of their adaptability to uneven skin surfaces and smoothness, polymer-based materials have been widely used in electrode fabrication. In one study, a self-adhesive PDMS nanofilm based electrode was fabricated using electrospinning and dip coating techniques [17]. In another, polyethylene terephthalate (PET) woven arrays were developed to serve as a backing for poly(3,4- ethylenedioxythiophene)-poly(styrenesulfonate) (PEDOT-PSS) coating [18]. PEDOT-PSS has also been printed on PET substrate, cotton fabrics, and paper using inkjet and screen printing technologies for ECG and photoplethysmography (PPG) measurement applications [19–21]. Metallic-based materials have also been widely used in wearable electronics because of their outstanding electrical properties. Silver has been screen printed on a TPU layer with multi-walled carbon nanotubes in a wearable shirt concept [22]. Resilient armbands have also been proposed aiming for suppressing motion-related artifacts [23]. In one study, silver-coated threads were sewn into a stretchable fabric [24]. Inkjet printing has also been exploited with silver for building electronics on fabrics enabling chemical reduction (i.e., reactive inkjet printing) [25]. Though not as prevalent as silver, gold has also been used with inkjet printing for ECG and EEG electrode applications [26–28].

Likewise, carbon-based materials, due to their electrical and mechanical properties, as well as their excellent chemical stability (e.g. graphene) [29] and large surface-to-volume ratio for functionalization, have also emerged as suitable candidates for electrode materials in biosensing and biopotential monitoring applications. In mobile healthcare applications, graphene has been used in the fabrication of conductive textile electrodes for monitoring of vital signs including electrocardiogram (ECG), electroencephalogram (EEG), electromyogram (EMG), and electrooculogram (EOG) [30, 31]. For instance, graphene-based textile electrodes were developed based on strategies like dip-coating and spray printing after which they were embedded in fashionable wearables (e.g., armband, sleep mask) [32, 33]; as well as, removable tattoos [34]. Innovative methods have also been proposed to realize printable graphene ink formulations [35], where some approaches including 3D printing and CO2 laser production techniques have been explicitly demonstrated for the fabrication of graphene-based ECG electrodes for humans [36] and animals [37] alike.

**Fig. 1:**
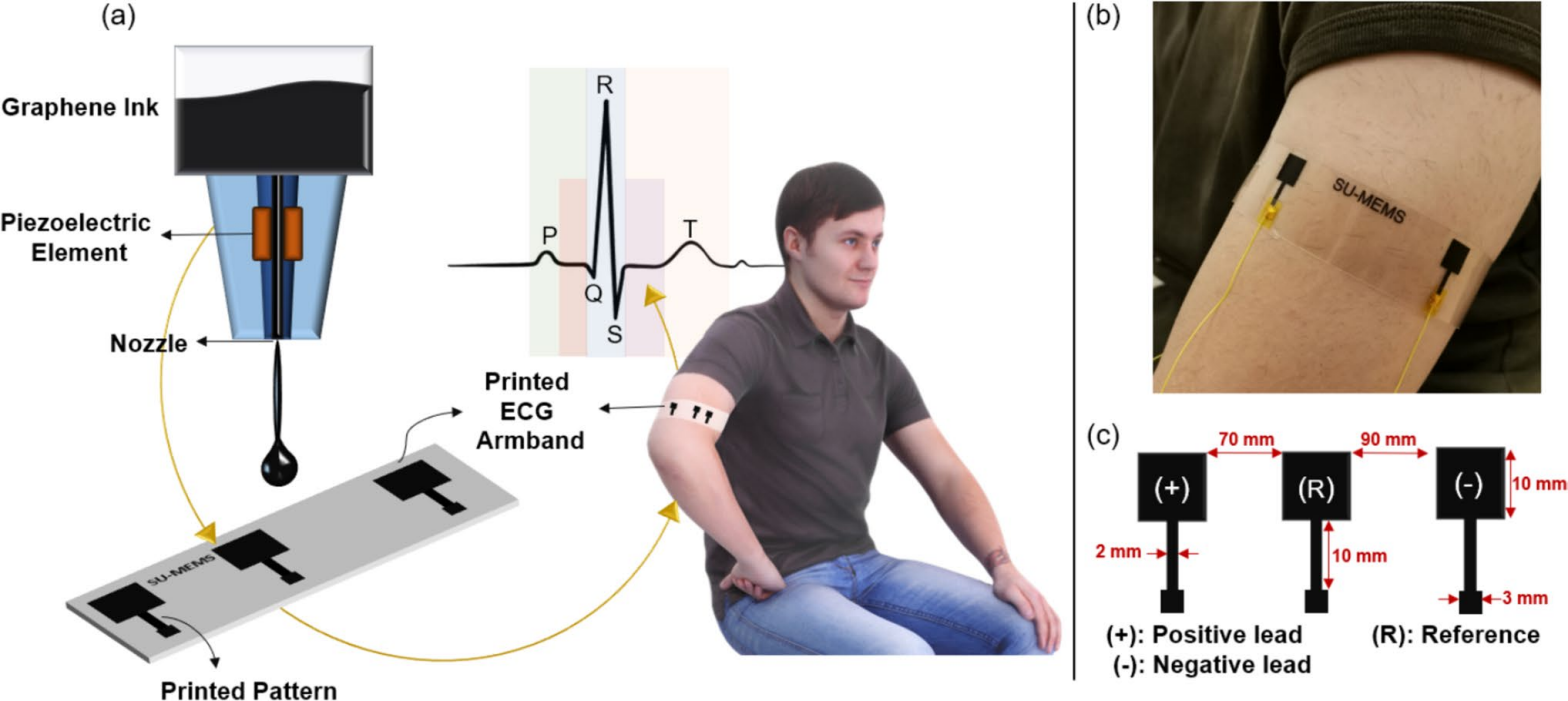
*Visual abstract of the study:* Printed electrodes are directly wired to the readout circuitry. Both the electrode size and the distance between the differential electrodes and the reference electrode can be adjusted during the printing process. **(a)** depicts the printing cycle. **(b)** shows the printed and annealed electrodes while recording ECG signals, and **(c)** shows the binary image design drawn in the printer’s interface

To the best of our knowledge, this is the first study to systematically employ a commercial inkjet printing platform for the fabrication of graphene-only ECG electrodes in a single, fully monolithic, patch-like armband configuration, with a dedicated focus on optimizing graphene jetting behavior to print biopotential electrodes for electrophysiological monitoring applications. The proposed device is fabricated as a “plug-and-measure” tri-electrode integrated structure on a plastic substrate and is ready for use immediately by electrical wiring to a data acquisition circuitry; without the need for post-assembly, placement of additional external electrodes or adjustment of electrode locations (Fig. 1). While prior studies have reported the use of graphene-based inks printed with inkjet systems [38, 39], their focus was not on a monolithic, wearable armband device, nor did they include single-channel armband ECG recordings or detailed ECG signal morphology analysis. Other inkjet-based studies have demonstrated ECG acquisition using metallic inks, such as silver, typically on polyimide substrates [40]; however, these approaches rely on non-monolithic electrode configurations and user-dependent hand placement to acquire lead-I ECG signals. Similarly, inkjet-printed ECG electrodes based on alternative conductive polymers, such as PEDOT:PSS, have been reported [41], yet without demonstrating a fully integrated, graphene-only, monolithic armband device. In contrast, the present work combines material selection, process optimization, and device integration to realize a ready-to-use, wearable ECG armband fabricated entirely through inkjet printing. The printed armband was wrapped around the arm of the wearer, and showed comparable performance in terms of ECG signal quality and usability to the standard Ag/AgCl ”wet” medical electrodes. The signal-to-noise ratio (SNR) scores in dB units were reported along with their impedance and signal energy scores. We demonstrate that the unique electrical and mechanical properties of graphene, combined with the cost-effective, large-scale, and direct maskless fabrication enabled by inkjet printing, offer strong potential for the development of next-generation mobile cardio-activity monitoring systems.

## Methodology

The armband was printed in a piezoelectric inkjet printer (Dimatix Materials Printer, DMP-2850, Fujifilm Inc. USA) equipped with a 2.4 pL drop volume cartridge. (Samba Dimatix Materials Cartridge, Fujifilm Dimatix Inc. USA). The electrode positioning is illustrated in Fig. 1c. All three electrodes, including the reference electrode, were printed on the same plastic sheet. The distance between the reference electrode and the first differential ECG electrode was 70 mm, while the distance between the reference electrode and the second differential ECG electrode was 90 mm.

Commercial water-based graphene ink (DMGRA-9003, Dycotec Materials Ltd, UK) was used to print the armband. The purchased graphene ink was diluted with a solution (DM-GRA-9003-DT, Dycotec Materials Ltd, UK) containing diethylene glycol and ethyl alcohol in an ink:diluent volume ratio of 1:0.9, resulting in a viscosity of 6.6 cps at 50 Pa and 25 $^{\circ }$C and a surface tension of 32.9 mN/m (Fig. S1, Supporting Information). The diluents were investigated for their epidermal toxicity. While ethyl alcohol completely evaporates from the electrodes during the annealing process, diethylene glycol poses no health problems in epidermal contact due to its much less effective transdermal absorption and its solid form in the printed electrodes [42, 43].

The solution ink was bath sonicated prior to printing for 30 minutes, followed by a degassing step in an ultrasonic cleaner (WF-UD,Weightlab Instruments, Turkiye) to ensure successful printing without nozzle clogging. The printing was performed at 30 Volts, 2.5 kHz frequency with a custom-designed waveform that enabled the desired jetting and drop formation. The jetted drop velocity was calculated as 4 m/s with the reported device settings. The drop spacing was set to 10 $\mu V$ to obtain optimum conductivity. While the temperature of the nozzles was set to 28 $^{\circ }$C, the platen that hosts the substrate was set to 40 $^{\circ }$C to make the solvent easy to evaporate during printing. PET film with 130 $\mu m$ thickness (HP Premium TransparencyFilm, Hewlett-Packard, USA) was used as the substrate and the distance between the printing head and the substrate was set to 1000 $\mu m$.

Multi-cycle printing was conducted with 5-minute intervals between each cycle, without removing the substrate from the inkjet printer. An annealing process at 120 $^{\circ }$C for 1 hour was performed on a hotplate to facilitate the evaporation of solvents and enhance electrical conductivity. The armband design has three separate electrodes with a sensing region of 1 x 1 cm each. The sensing region is linked to an adjacent square where a connection to the read-out circuitry was made and secured through a 2 mm $\times$ 10 mm stripe as shown in Fig. 1. To benchmark the performance of the electrodes, two sets of ECG data were collected with the printed graphene sensors and the commercial Ag/AgCl electrodes asynchronously at adjacent locations of the left arm. An open-source bio-potential acquisition unit (Cyton Board,OpenBCI) was used with no analog filtering circuit for data collection. The raw data was processed digitally in custom written MATLAB scripts (MathWorks, Natick, Massachusetts, USA). The powerline interference at 50 and 100 Hz was first removed from the normalized ECG signals using a notch filter with a Q factor of 35. Then, a bandpass filter was applied to suppress the noise below 5 Hz and above 50 Hz. Finally, a fifth-order moving average filter was used for smoothing. A digital LCR meter (LCR-6002, GW Instek, Taiwan) was used to measure skin-electrode impedance. For this purpose, the electrodes were placed on the forearm with 5 cm spacing, and the skin was cleaned with alcohol prior to electrode placement. The printed graphene electrodes were positioned on the forearm with clamps to ensure consistent applied pressure. Measurements were carried out between 10 Hz and 1 kHz, and the collected data were normalized by multiplying by the surface area of the electrodes to account for differences in electrode size.

## Results and Discussion

To assess the usability of the printed sensors, they were first benchmarked against commercial Ag/AgCl electrodes. Before printing them in their final side-by-side configuration on a plastic armband, individual sensors were initially printed and tested separately by attaching them one by one to the participant’s left arm for preliminary trials. The signal-to-noise ratio (SNR) metric in dB units was used to compare the signal qualities, where the energy around the QRS complex represents the signal and the outer fluctuations between consecutive S- and Q- containing P-, T- waves, and isopotential lines represent the noise (Eq. 1). The same rule applies when computing RMS noise.

1 $$\begin{aligned} {SNR [dB] } = 20 \, log \Bigl ( \frac{ | E_{rms,QRS} | }{E_{noise}} \Bigl ) \end{aligned}$$

At this stage, it is important to emphasize that the signal quality metric employed in this study calculates the ratio of signal energies around the QRS complex relative to the isopotential line. All SNR calculations are performed after the signal has been preprocessed using basic digital filtering methods, including bandpass filtering and moving average smoothing, to reduce noise and baseline drift. Moreover, the metric is sensitive to variations in experimental conditions -such as changes in skin properties (e.g., perspiration) or even slight shifts in electrode placement- which can significantly affect the amplitude of the QRS complex and thus the measured energy. Therefore, this metric is most appropriately used for comparing different electrode types, such as printed graphene electrodes versus commercial wet electrodes, when experiments are conducted simultaneously or in quick succession. It is less suitable for comparisons across trials performed under differing conditions or at separate times and locations. In summary, considering the nature of the SNR calculation described above, the results should not be interpreted as conclusive evidence that one electrode type outperforms the other. Rather, comparable SNR scores suggest that the two types exhibit similar performance and may be considered viable alternatives under the tested conditions.

**Fig. 2:**
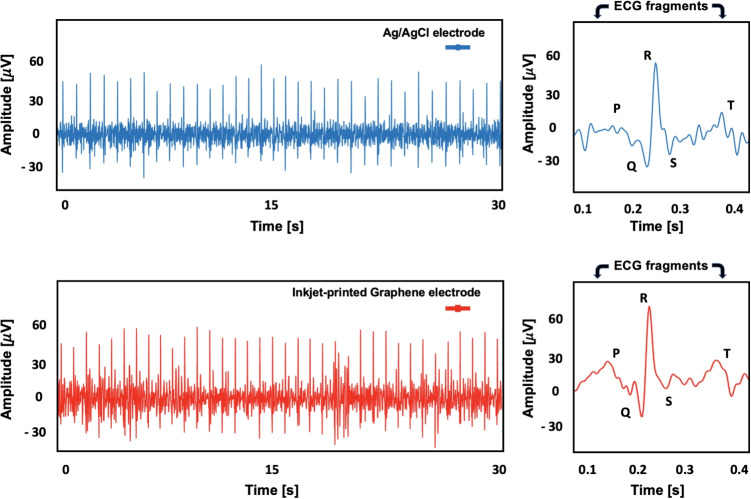
Two ECG recordings were obtained subsequently from the same participant at the exact same location of the left arm. Printed sensors have provided an SNR score of 5.12 dB

Moving on to the first phase of the experiments, Fig. 2 presents two ECG recordings obtained from commercial and printed sensors, respectively. The data were collected asynchronously, with both types of electrodes placed at the exact same location during separate trials. The signals shown are temporal 30-second windows. The experiments were conducted under static conditions to perform precise benchmarking. The subject (31 years old, no CVD history) was instructed to sit still in a chair to avoid involuntary bicep muscle contraction and signal interference. Due to asynchronous measurements made in this phase of the study, the P-QRS-T complexes in both recordings are not identical; thus, we did not present a correlation score. Instead, a quality score along with peak-to-peak voltage would present a reliable comparison between inkjet printed sensors and gold standard Ag/AgCl electrodes. While the SNR value obtained from the commercial electrodes was 4.46 dB with a mean peak-to-peak voltage of Vpp=103 $\mu V$, graphene-based sensors provided an SNR score of 5.12 dB with peak-to-peak voltage of Vpp=109 $\mu V$.

In the second phase of the study, four participants (aged 25-34, all healthy with no history of cardiovascular disease) were recruited for a more detailed trial aimed at investigating signal morphology through side-by-side, synchronous data collection. The same printed armband was used to record ECG signals from all participants. To enable accurate assessment of correlation ratios, commercial Ag/AgCl electrodes were placed in close proximity to the printed sensors - so close that the adhesive surface of the wet electrodes was in direct contact with the plastic of the armband. Table 1 shows the SNR quality scores for each participant, with each row corresponding to an individual. Each participant has two datasets: one recorded using a plastic graphene inkjet-printed armband, and the other using commercial wet Ag/AgCl electrodes. The table also includes the correlation ratios between the signals captured by the two electrode types. As can be seen, even in the worst case, the correlation reaches at least 80-85%, which reflects a strong similarity in signal shape and timing - particularly considering the use of different electrode materials and designs. While some differences do exist, these are minor and expected. They can largely be attributed to the fact that, although the electrodes were placed in close proximity to ensure synchronized data acquisition, it is not physically possible to place them in the exact same location. This slight spatial offset leads to small variations in signal quality, which is normal and consistent with previous findings in electrophysiological recordings [44]. For Participant 1, the correlation ratio is 96.21%, with an SNR difference of about 4 dB. Participant 2 shows a correlation of 98.37%, with SNR scores that are nearly identical between the two electrode types. Participant 3 has the lowest correlation at 89.88%, and Participant 4 shows a correlation of 96.77%. In addition to SNR and correlation scores, mean RSS values were also reported to provide insight into the nature of noise present in the signals. Specifically, mean RSS was calculated around the QRS complex -representing the ”signal” component in the SNR calculation - and along the isopotential line, representing ”noise.” The largest difference in mean RSS was observed for Participant 4, with a gap of approximately 20 $\mu$V. The smallest difference was seen in Participant 1, where the mean RSS values differed by only 7 $\mu$V. Overall, the results indicate that while slight SNR differences exist between graphene inkjet-printed electrodes and commercial Ag/AgCl electrodes, the high correlation and similar signal quality scores suggest that the inkjet-printed electrodes are a viable alternative to traditional wet electrodes for biosignal acquisition.

**Table 1:** Presents the comparative results between graphene inkjet-printed electrodes and commercial Ag/AgCl electrodes. Each row corresponds to a participant, with two data sets provided: one for the inkjet-printed electrodes and the other for the commercial electrodes. In addition to correlation ratios and SNR scores, mean RSS values for both ”signal” and ”noise” components are included to give the reader insight into the characteristics of the noise and overall signal quality

Feature $\rightarrow$		Correlation Ratio	SNR Score	RMS Noise	Mean RSS around	Mean RSS between
Configuration $\downarrow$			[dB]	$[\mu V]$	QRS complexes	QRS complexes
					$[\mu V]$	(noise) $[\mu V]$
Participant #1	Printed Graphene	% 96.21	11.99	1.20	48.78	12.43
	Ag/AgCl		7.79	2.36	41.88	18.15
Participant #2	Printed Graphene	% 98.37	10.30	1.55	61.58	18.79
	Ag/AgCl		10.87	1.38	50.16	14.34
Participant #3	Printed Graphene	% 89.88	4.32	1.53	34.87	18.88
	Ag/AgCl		4.01	0.78	16.34	9.46
Participant #4	Printed Graphene	% 96.77	2.67	2.97	58.24	26.95
	Ag/AgCl		3.54	4.04	38.27	46.41

**Fig. 3:**
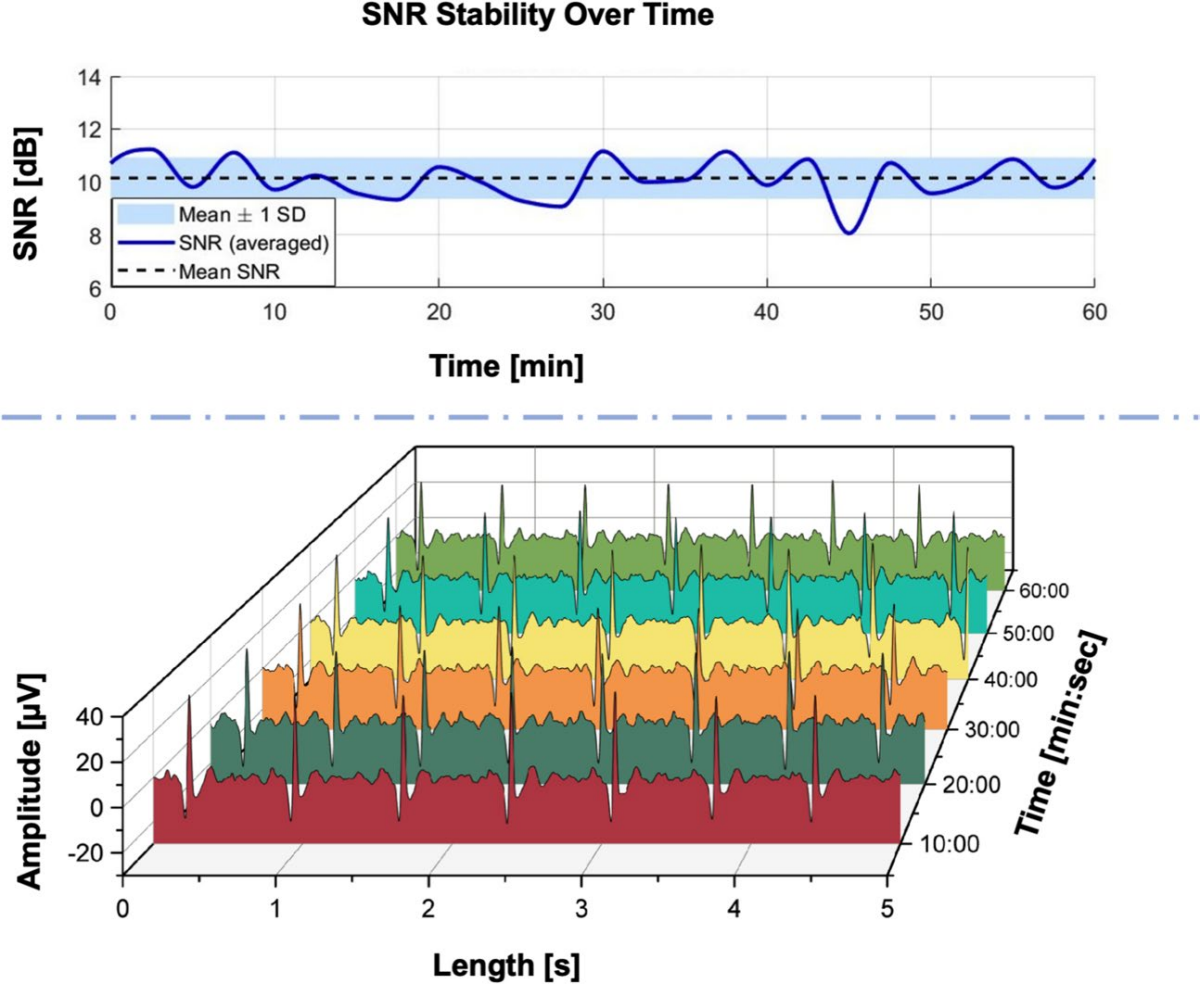
The results of the long-term measurement experiment, in which a participant wore the proposed armband while sitting still in front of a computer screen for over an hour, are presented. The SNR stability graph, shown above the amplitude graph, illustrates the consistent performance of the armband. The SNR remained within a standard deviation of only 0.78, with a relative standard deviation of just 7.7%

Another important aspect of assessing the reliability of a dry electrode is its long-term performance. In the third phase of this study, we evaluated the long-term measurement capability of the graphene-printed plastic armband by recording a one-hour ECG session with our final participant, Participant #4 (28 year old, male, no CVD). Notably, the armband used in this test was printed in three cycles, rather than a single one. The participant was instructed to sit still in front of a computer screen and to watch an hour-long TV series episode in his preference, where he occasionally moves his arms voluntarily to relieve his muscles. A pair of commercial Ag/AgCl electrodes were placed adjacent to the graphene sensors. Figure 3 illustrates six sample sets extracted from one hour of ECG data. The SNR values plotted in the graph were calculated approximately every two minutes and then averaged. They range from 8.04 to 11.23, with a mean of approximately 10.14, represented by a dashed line in the middle of the graph. The blue shaded area denotes the standard deviation, which is around 0.78, indicating modest variability in signal quality over time. To provide further context, the relative standard deviation (RSD) was calculated to be 7.7%, suggesting a relatively stable signal with minor fluctuations. This low RSD supports the reliability of the recording system for long-term use, making it well-suited for continuous monitoring applications.

The third phase of the study aimed to evaluate motion sensitivity under real-world conditions and to identify potential motion-induced artifacts. A participant was asked to perform everyday activities while wearing the plastic inkjet-printed graphene armband, including sitting still at a computer, walking casually, and performing squats similar to a gym exercise. SNR values were recorded throughout each activity. The experiments were conducted in a controlled laboratory environment. The participant was provided with an 18-square-meter empty room at ambient room temperature and wore a very short-sleeved T-shirt during all tasks. While sitting still, the average SNR was measured at 4.63 dB, with an RMS noise value of 3.60 $\mu$V. During casual walking, the average SNR dropped to 1.55 dB, and RMS noise increased to 6.77 $\mu$V. After a short rest, the participant performed squats, resulting in a further decrease in SNR to 0.74 dB and an RMS noise of 7.65 $\mu$V. These reductions in signal quality were expected under motion, yet the QRS complexes remained distinguishable in the ECG waveform, even during squatting. In future work, we plan to explore advanced signal processing techniques, including regenerative filtering methods, to improve motion robustness and enhance the reliability of the ECG collection system. For this benchmarking study, only basic signal conditioning was applied - specifically, passive bandpass and notch filters, along with a simple moving average for smoothing.

**Fig. 4:**
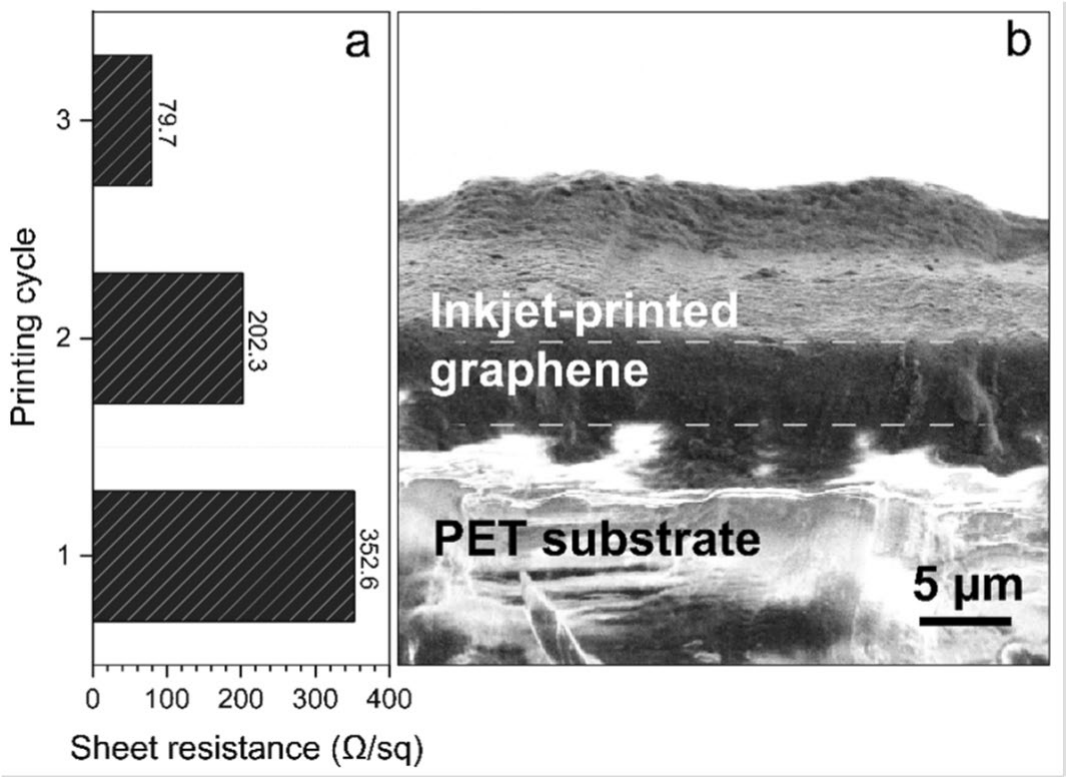
(**a**) Sheet resistance measurements of graphene electrodes printed with varying cycles of 1, 2, and 3. (**b**) Cross-sectional SEM profile of the 3-cycle printed graphene on a PET substrate

The fourth phase of the study involves measuring the electrical properties of the graphene electrodes. To quantitatively characterize the performance of the electrodes printed with different numbers of printing cycles (1, 2, and 3), the sheet resistance was measured for separate samples fabricated using each cycle count (Fig. 4). A four-point probe system (Microtech CP4) was utilized to assess the sheet resistance, with current supplied through the external probes and the resulting voltage drop measured across the internal probes. The results indicate that electrical conductivity increased with the number of printing cycles. Specifically, the sheet resistance values were 352.6 $\Omega$/sq for one cycle, 202.3 $\Omega$/sq for two cycles, and 79.7 $\Omega$/sq for three cycles. This consistent decrease in sheet resistance is attributed to the increased thickness of the printed graphene, which enhances electrical conductivity. Figure 4 illustrates the scanning electron microscopy (SEM) image of cross-sectional profile of the 3-cycle printed graphene on a PET substrate, with a uniform coverage of graphene and estimated graphene thickness of 5.32 ± 0.64 $\mu$m.

**Fig. 5:**
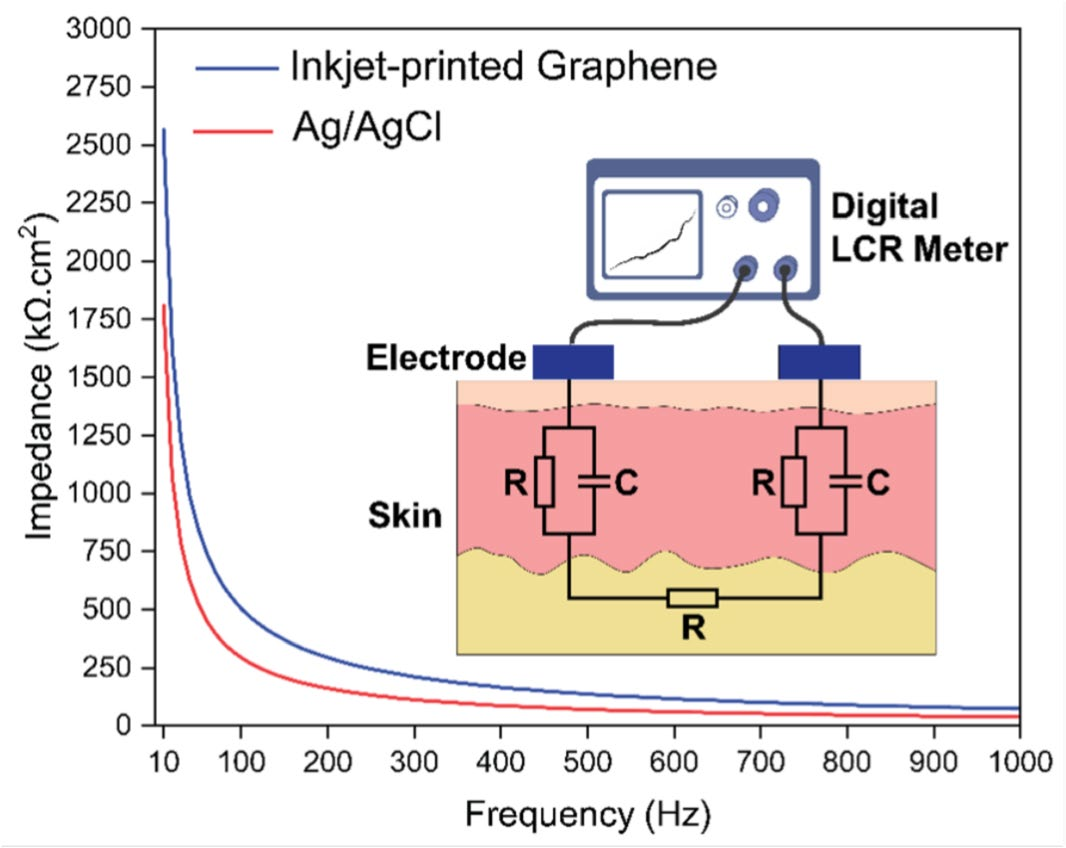
Area-normalized skin-electrode impedance of commercial gel-based Ag/AgCl and inkjet-printed graphene electrodes across a frequency range of 10 Hz to 1 kHz. The results demonstrate relatively higher impedance values for the graphene dry electrodes compared to the Ag/AgCl electrodes with conductive gels

Moving on to the assessment of electrical properties, another important factor is the skin–electrode impedance. This is a key indicator of the effectiveness of biopotential electrodes in recording biosignals. Low skin-electrode impedance is associated with high SNR signals and reduced susceptibility to motion artifacts [45]. Figure 5 demonstrates the area-normalized impedance values of gel-based Ag/AgCl and printed graphene electrodes across a frequency range of 10 Hz to 1 kHz. As shown, the normalized impedance values of conventional Ag/AgCl and graphene electrodes at 10 Hz are 1809 and 2566 k$\Omega$ $\cdot$cm$^{2}$, respectively. To demonstrate the consistency of the measured impedance values across different demographic markers, the impedance was measured in three participants (Fig. S2, Supporting Information). The results show relatively higher values of normalized skin-electrode impedance in printed graphene electrodes compared to Ag/AgCl electrodes. This difference arises from the absence of a conductive gel layer, which effectively enhances the skin-electrode interface contact and thus reduces the impedance value.

Although single-arm ECG is inherently limited in capturing comprehensive cardiovascular dynamics due to the difficulty of extracting deep physiological insights from just one signal channel, it remains a key focus in many wearable health monitoring studies. Many have investigated optimal electrode placement on the arm [46], and a wide range of armband sensor designs have been proposed over the years. For instance, some researchers have embedded capacitive-coupled electrodes into textile-based armbands, testing their performance under real-world conditions such as jogging [47]. These systems reported peak detection error rates of around 8% . Other works have developed fully integrated solutions using sintered silver–silver chloride dry electrodes, showing that low-level physical activity can reduce SNR by approximately 3–4 dB and increase R-peak detection error rates by up to 1.9% [48]. Alternative materials have also been explored. One group utilized carbon-based sensing elements and, instead of traditional SNR metrics, analyzed signal quality using measures like entropy and skewness [49]. Their comparative study demonstrated that usable ECG data could be captured during roughly 75% of non-bedtime hours and nearly 100% during rest, with a reported 10% performance gap relative to the Holter device - though without a direct electrode-to-electrode comparison. This proposal was later adapted for atrial fibrillation detection [50]. Graphene-based textile armband sensors have also shown promise, with studies reporting SNR degradation and up to a 9% drop in correlation with commercial Ag/AgCl electrodes during light physical activity such as walking [44]. These developments underscore the continued momentum in armband-based ECG research. While diagnostic limitations remain, particularly in clinical-grade applications, the simplicity, comfort, and accessibility of armband systems -especially for individuals with limited mobility- make them a compelling tool for long-term personal healthcare monitoring. Innovations in electrode materials, such as silver compounds or graphene composites, are likely to expand their capabilities even further.

## Conclusion

Non-disposable bio-potential electrodes make it feasible to track patient cardio-activities in clinical and non-clinical (e.g. home) settings. The ease of clinical measurements in home settings would make a significant impact on managing chronic diseases, particularly for vulnerable populations like the elderly and children. For many years, researchers have proposed reliable, real-time, and affordable cardio-monitoring systems, including invasive and non-invasive solutions where it is easy to track the body’s bio-potential differences. With this work, we propose novel insights into the long-term ECG measurement literature by incorporating inkjet technology and the outstanding characteristics of graphene material. We successfully demonstrated the usability of easy-to-produce graphene electrodes on different participants and showed the excellent potential of inkjet printing for future digital healthcare applications. In summary, the printed electrodes demonstrated ECG signal quality on par with commercial Ag/AgCl electrodes across diverse conditions -including rest, walking, and squatting- highlighting their potential for reliable, motion-tolerant physiological monitoring in real-world applications.

## Supplementary Information

Below is the link to the electronic supplementary material.

Supplementary file 1 (pdf 650 KB)

## Data Availability

No datasets were generated or analysed during the current study.
