# Supplementary material for: Inkjet-Printed Graphene Electrodes on a Plastic Armband for Mobile Electrocardiography
Source: J Med Syst. 2026 Mar 5;50(1):27. doi: 10.1007/s10916-026-02357-6 (PMC12960339; doi:10.1007/s10916-026-02357-6)
Supplement: Supplementary file 1 — (pdf 650 KB) [file 10916_2026_2357_MOESM1_ESM.pdf]

Inkjet-Printed Graphene Electrodes on a Plastic Armband for Mobile Electrocardiography

Saygun Guler[1†], Seyed Sajjad Mirbakht[1†], Melih Can Tasdelen[1], Burcu Arman Kuzubasoglu[1,2], Faruk Ballipinar[1,2], Murat Kaya Yapici[1,2,3*]

[1]Faculty of Engineering and Natural Sciences, Sabanci University, Orta Mah., Universite Cad., Istanbul, 34956, Türkiye.
[2]Sabanci University Nanotechnology Research and Application Center (SUNUM), Sabanci University, Orta Mah., Universite Cad., Istanbul, 34480, Türkiye.
[3]Department of Electrical and Computer Engineering, University of Washington, 185 Stevens Way, Seattle, Washington, 98195-2500, USA.

*Corresponding author(s). E-mail(s): mkyapici@sabanciuniv.edu;
Contributing authors: saygun.guler@sabanciuniv.edu; seyed.mirbakht1@sabanciuniv.edu; mctasdelen@sabanciuniv.edu; burcu.arman@sabanciuniv.edu; ballipinar.faruk@gmail.com;
†Equal contribution

1 Supplementary Material

Please refer to the next page for the supplementary figures.

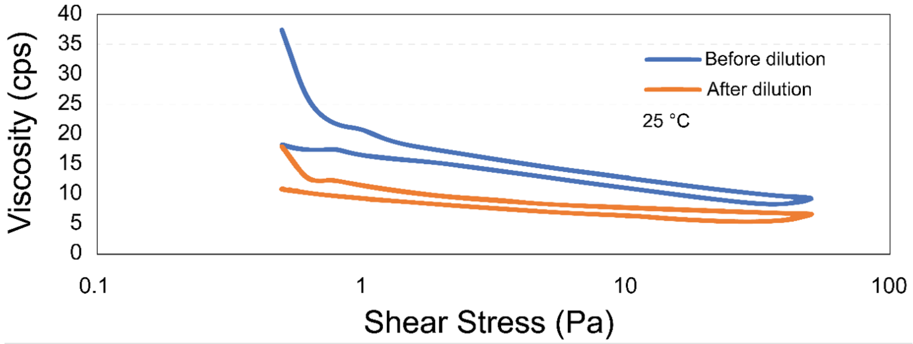

Fig. 1 Flow curve of graphene ink before dilution and after dilution with diethylene glycol and ethyl alcohol in an ink:diluent volume ratio of 1:0.9. As shown, the viscosity decreased from 9.2 cps to 6.6 cps at 50 Pa and 25 °C after dilution.

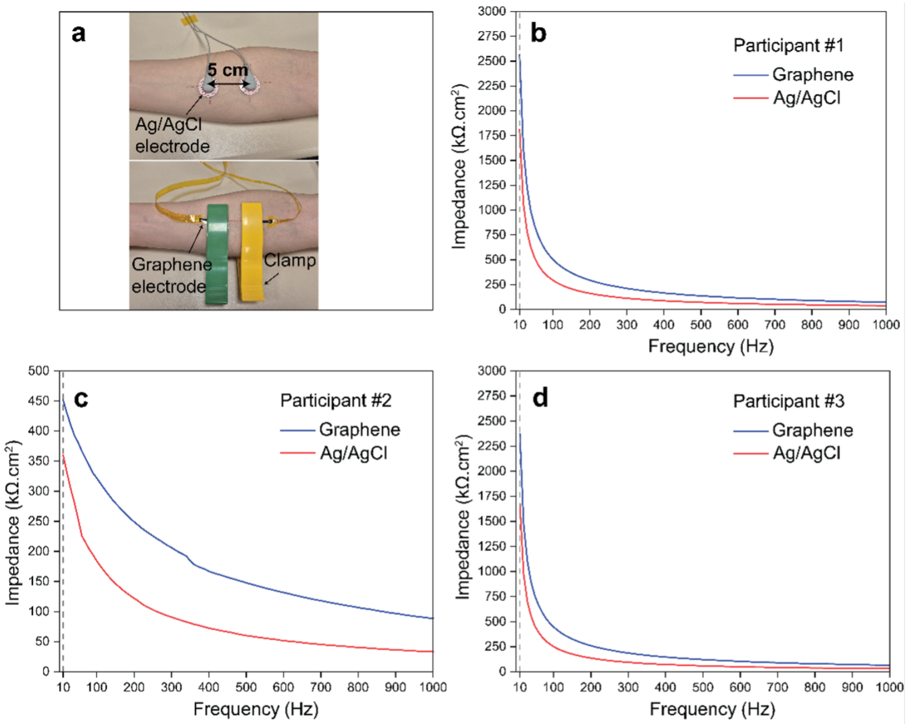

Fig. 2 a) Measurement setup for skin-electrode impedance. The Ag/AgCl and printed graphene electrodes were placed on the participant's forearm with 5 cm spacing. The graphene electrodes were positioned with clamps to ensure consistent applied pressure across all participants. b, c, and d) Area-normalized skin-electrode impedance over three participants, showing relatively higher values for graphene electrodes compared to Ag/AgCl gel-based electrodes.
